# Activation of nuclear factor-*κ*B in human prostate carcinogenesis and association to biochemical relapse

**DOI:** 10.1038/sj.bjc.6602851

**Published:** 2005-11-08

**Authors:** J Domingo-Domenech, B Mellado, B Ferrer, D Truan, J Codony-Servat, S Sauleda, J Alcover, E Campo, P Gascon, A Rovira, J S Ross, P L Fernández, J Albanell

**Affiliations:** 1 Department of Medical Oncology and Laboratory of Experimental Oncology (ICMHO), Hospital Clinic & Institut d'Investigacions Biomediques August Pi i Sunyer (IDIBAPS), Barcelona, Spain; 2 Department of Pathology, Hospital Clinic & Institut d'Investigacions Biomediques August Pi i Sunyer (IDIBAPS), Barcelona, Spain; 3 Departments of Urology, Hospital Clinic & Institut d'Investigacions Biomediques August Pi i Sunyer (IDIBAPS), Barcelona, Spain; 4 Blood Bank Center, Hospital Vall d'Hebron, Barcelona, Spain; 5 Department of Pathology and Laboratory Medicine, Albany Medical College, Albany, NY, USA; 6 Millennium Pharmaceuticals, Inc., Cambridge, MA, USA

**Keywords:** prostate cancer, NF-*κ*B, transcription factor, malignant transformation, PSA

## Abstract

Nuclear factor (NF)-*κ*B/p65 regulates the transcription of a wide variety of genes involved in cell survival, invasion and metastasis. We characterised by immunohistochemistry the expression of NF-*κ*B/p65 protein in six histologically normal prostate, 13 high-grade prostatic intraepithelial neoplasia (PIN) and 86 prostate adenocarcinoma specimens. Nuclear localisation of p65 was used as a measure of NF-*κ*B active state. Nuclear localisation of NF-*κ*B was only seen in scattered basal cells in normal prostate glands. Prostatic intraepithelial neoplasias exhibited diffuse and strong cytoplasmic staining but no nuclear staining. In prostate adenocarcinomas, cytoplasmic NF-*κ*B was detected in 57 (66.3%) specimens, and nuclear NF-*κ*B (activated) in 47 (54.7%). Nuclear and cytoplasmic NF-*κ*B staining was not correlated (*P*=0.19). By univariate analysis, nuclear localisation of NF-*κ*B was associated with biochemical relapse (*P*=0.0009; log-rank test) while cytoplasmic expression did not. On multivariate analysis, serum preoperative prostate specific antigen (*P*=0.02), Gleason score (*P*=0.03) and nuclear NF-*κ*B (*P*=0.002) were independent predictors of biochemical relapse. These results provide novel evidence for NF-*κ*B/p65 nuclear translocation in the transition from PIN to prostate cancer. Our findings also indicate that nuclear localisation of NF-*κ*B is an independent prognostic factor of biochemical relapse in prostate cancer.

Nuclear factor (NF)-*κ*B/Rel transcription factors represent a group of structurally related proteins, with five members in mammals, including p65 (also known as RelA) (Baldwin, 1996; Ghosh *et al*, 1998; Karin *et al*, 2004). Nuclear factor-*κ*B is rendered inactive in nonstimulated cells in the cytoplasm due to its association to NF-*κ*B inhibitory proteins, known as I*κ*Bs. The activation of NF-*κ*B, and in particular of p65, results from the polyubiquitinination and subsequent 26S proteasome degradation of I*κ*Bs, which allows then the translocation of p65 to the nucleus. Nuclear p65 initiates the transcription of a wide variety of genes that code for angiogenic factors, cell adhesion molecules, antiapoptotic factors and cytokines, which are involved in cell survival, tumour invasion and metastasis. Extensive studies performed on cancer cell lines and preclinical models support a role of NF-*κ*B in cancer development and progression (Karin *et al*, 2002). Recently, a number of studies in human solid tumours and haematological malignancies have also provided clinical data to support the concept that NF-*κ*B may have an important role *in vivo* in human cancer (Rayet and Gelinas, 1999; Karin *et al*, 2002). The importance of further characterisation of NF-*κ*B expression in clinical cancer specimens is highlighted by the current efforts to develop novel drugs that inhibit NF-*κ*B activation with the ultimate goal to use them for cancer treatment (Orlowski and Baldwin, 2002; Karin *et al*, 2004).

Prostate cancer is the second leading cause of death in men from cancer. Despite the availability of local treatment with radical prostatectomy or radiotherapy, which are often curative, many patients develop disease relapse after primary therapy. The first sign of prostate cancer recurrence is often heralded by an increase of serum prostate-specific antigen (PSA) levels and is known as biochemical relapse. Subsequently, patients can develop clinical relapse of the disease typically featuring bone metastases (Pound *et al*, 1999). It is widely held that the discovery of novel biomarkers designed to predict the risk of relapse or that could be used as targets of new targeted anticancer agents are clearly needed (Ross *et al*, 2003). Along this line, several preclinical observations have shown that NF-*κ*B plays a role in prostate cancer growth, survival, angiogenesis and metastatic progression (Muenchen *et al*, 2000; Yu *et al*, 2000; Catz and Johnson, 2001; Huang *et al*, 2001; Gasparian *et al*, 2002; Gunawardena *et al*, 2002; Gupta *et al*, 2002; Suh *et al*, 2002; Zerbini *et al*, 2003; Suh and Rabson, 2004). Furthermore, emerging data obtained from human studies also suggest a potential role of NF-*κ*B in prostate cancer tumorigenesis and/or in its behaviour in a clinical setting (Suh *et al*, 2002; Lessard *et al*, 2003; Fradet *et al*, 2004; Ismail *et al*, 2004; Ross *et al*, 2004). In particular, a recent study showed that total overexpression of p65, the best characterised NF-*κ*B protein, was an independent predictor of poor prognosis in prostate cancer patients (Ross *et al*, 2004). Of note in this study was the fact that nuclear NF-*κ*B expression was not specifically linked with disease relapse. However, in another study performed in patients with prostate cancer and pathologically positive surgical margins in the prostatectomy specimens, NF-*κ*B nuclear localisation was associated to a high risk of biochemical relapse (Fradet *et al*, 2004).

In the present study, we characterised by immunohistochemistry (IHC) the expression and subcellular localisation patterns of NF-*κ*B in normal prostate, prostatic intraepithelial neoplasia (PIN) and prostate adenocarcinoma (PAC). The results revealed that NF-*κ*B/p65 nuclear translocation occurs in the transition of PIN to prostate cancer. We also assessed whether nuclear NF-*κ*B expression (used as a measure of the active state of NF-*κ*B), when separately analysed, predicts biochemical relapse in 86 PAC patients. The results of this series, the largest reported to date, confirm a link between NF-*κ*B and prostate cancer outcome and extend prior reports by showing that nuclear NF-*κ*B (the biologically active form) is the strongest independent prognostic factor of biochemical relapse in men that underwent prostatectomy as assessed by a comprehensive multivariate analysis. A combined prognostic analysis taking into account preoperative PSA levels, Gleason grade and nuclear localisation of NF-*κ*B staining identified subsets of patients with markedly different risks of biochemical relapse.

## MATERIAL AND METHODS

### Specimen collection and patients

A total of 105 tissue specimens were selected for the present study following institutional guidelines. Paraffin blocks containing sufficient formalin-fixed tissue for marker analyses were obtained from six histologically normal prostate specimens, 13 cases of high grade PIN, and 86 PACs. The selected tissues were obtained from 86 radical prostatectomy specimens that were performed for biopsy-proven PAC at the Hospital Clinic of Barcelona. The normal prostatic tissues and high-grade PIN lesions came from the same patients with from whom prostate cancer was assessed. All clinical follow-up studies were also performed at the Hospital Clinic. This study was part of a project funded by the Spanish Science and Technology Ministry (MCYT; Grant Number SAF 2003–08181) that had been approved by the Ethics Committee of our institution.

### Clinico-pathological data of prostate cancer patients

Tumour Gleason grading (Gleason, 1992), preoperative PSA serum levels, surgical margins, lymph-node status, seminal vesicle invasion, and pathological staging (Ohori *et al*, 1994) were collected from the 86 prostate cancer patients. Gleason scores were classified as high grade when the combined Gleason score was ⩾7 and low grade when the combined Gleason score was ⩽6 (Ross *et al*, 2004). Serum PSA was measured with Access Hybritech PSA assay (Beckman, San Diego, CA, USA). Serum PSA levels were obtained from patients prior to prostatectomy and classified as low when PSA levels were <10 ng ml^−1^ and high when PSA levels were ⩾10 ng ml^−1^ (Ross *et al*, 2004). An elevation of PSA from the baseline level post-prostatectomy to >0.4 ng ml^−1^ on two consecutive occasions was considered to be biochemical evidence of disease relapse. Surgical margins were defined as positive or negative. Pathologic stage was T2a, 14 (16.3%) patients; T2b, 37 (43.0%) patients, T3a, 22 (25.6%) patients; and T3b, 13 (15.1%) patients. Pathologic stage was classified as organ-confined (stage T2a and T2b) and advanced tumour (stage T3a and T3b) (Ross *et al*, 2004). Seminal invasion was classified absent in 73 (84.8%) tumours and present in 13 (15.2%). Lymph-node status was negative for metastatic disease in 81 (91.1%) patients and positive in 5 (5.9%). Follow-up data was obtained from review of the patients' medical records.

### Tissue microarray

Tissue microarrays were constructed following standard methodology. After selection of donor areas by microscopic examination, 2 mm punches were placed in a receptor block measuring 27 × 30 mm. Each microarray block contained a maximum of 80 punches and duplicates of each tumour were produced. Prior to sectioning, the surface of each microarray block was briefly heated on a hot plate to improve adhesion of the cores within the block. Each block was serially sectioned at 2–4 *μ*m using a Microm HM330 microtome. The sections were floated out on a water bath at 45°C and picked up onto sequentially numbered slides (DAKO). The slides were dried at 37°C overnight prior to staining. Standard sections were also taken from original tumour blocks in selected specimens to compare the immunohistochemical findings of the microarray sections with those of standard (larger) sections.

### Immunohistochemistry

Briefly, 2–4 *μ*m formalin-fixed paraffin-embedded sections from tissue microarray blocks were deparaffinised and rehydrated. Antigen retrieval was performed by heating slides in a pressure cooker in citrate buffer pH 6 for 5 min. The primary antibody, an IgG1 class rabbit polyclonal antibody against the carboxy terminus of p65/RelA component of the NF-*κ*B complex (sc-372, C-20, Santa Cruz Biotechnology Inc., Santa Cruz, CA, USA) was used at a dilution of 1 : 180. The slides were incubated with the primary antibody for 60 min, washed in ChemMate buffer solution (DAKO) and developed using the Envision signal detection system and the Techmate 500 equipment (DAKO Corp., Glosstrup, DM). To confirm the specificity of the primary antibody, immunoreactivity was blocked by preabsortion of the primary antibody with an excess amount of p65 antigen peptide (sc-372P, Santa Cruz Biotechnology Inc., Santa Cruz, CA, USA).

Immunoreactivity for NF-*κ*B was evaluated in normal prostate, PIN and PAC. Nuclear factor-*κ*B staining was semiquantitatively assessed as + (low staining), ++ (moderate staining) and +++ (strong staining), essentially following criteria previously reported by one of us (JSR). After initial assessments, PIN lesions had strong staining for p65 and cells of normal glands a weak staining. This information was used as the basis for further scorings. Specimens with +++ scoring had an intensity of staining similar to that observed in PIN lesions and specimens with + scoring had an intensity of staining similar to that observed in histologically normal prostatic epithelial cells. The ++ scoring was used for tumour cells, which expressed an intermediate degree of intensity. The percentage of cytoplasmic and nuclear staining was obtained from the average of the positive staining cells obtained in duplicate cores.

Staining of NF-*κ*B in surrounding inflammatory cells and endothelial cells served as internal positive control signals. At least two investigators, blinded to clinical data, scored each sample. Concordance between investigators for nuclear NF-*κ*B staining was >95%. A third investigator reviewed discordant cases.

### Immunofluorescence assay

The ability of the anti-p65 antibody used in IHC assays (sc-372, C-20; Santa Cruz) to detect nuclear translocation/activation of NF-*κ*B/p65 was tested by immunofluorescence in cultured cells. We performed the immunofluorescence experiment as a part of the validation process of the anti-p65 antibody to be used in IHC. The human prostate cancer cell line (PC-3) was grown in F-12K Nutrient Mixture (Gibco, California) medium supplemented with 10% foetal bovine serum. Cells were seeded in 35-mm tissue culture plates on sterile glass coverslips at a cell density of 200 000 cells per plate. Cells were treated with tumour necrosis factor (TNF)-*α* 100 ng ml^−1^ (CalBiochem, California) during 10 min. Cells were then fixed with methanol and washed with PBS. After blocking with PBS plus BSA 1%, cells were incubated with the primary anti-p65 antibody at a dilution of 1 : 180 during 2 h at 37°C. Then cells were washed with PBS and incubated with the secondary antibody Alexa Fluor 594-coupled goat-antirabbit IgG at a dilution of 1 : 1000 during 1 h at 37°C. The slides were further washed with PBS and then mounted with Mowiol (CalBiochem) for fluorescent microscopic examination. Fluorescence confocal and phase images were acquired using a Leica TCS SL laser scanning confocal spectral microscope (Leica Microsystems, Heidelberg GmbH, Manheim, Germany). Image assembly and treatment were performed using the Image Processing Leica Confocal Software.

### Statistical analysis

Statistical analysis was carried out with SPSS version 11.0 (SPSS, Inc., Chicago, IL, USA). To analyse correlations between p65 expression and other clinico-pathological variables, we used Spearman's correlation tests when the two variables were assessed as continuous, *t*-test when one variable was assessed as continuous and the other as qualitative and *χ*^2^ test (Fisher exact test) when the two variables were qualitative. Biochemical disease recurrence was analysed by the Kaplan–Meier method. Curves were compared by the log-rank test. Multivariate analysis including continuous quantitative and qualitative clinicopathological parameters was performed using the Cox proportional hazards model. All the statistical tests were conducted at the two-sided 0.05 level of significance.

## RESULTS

### Immunohistochemical expression of NF-*κ*B in histologically normal prostate, prostate intraepithelial neoplasia and prostate cancer tissues

**Figure 1:**
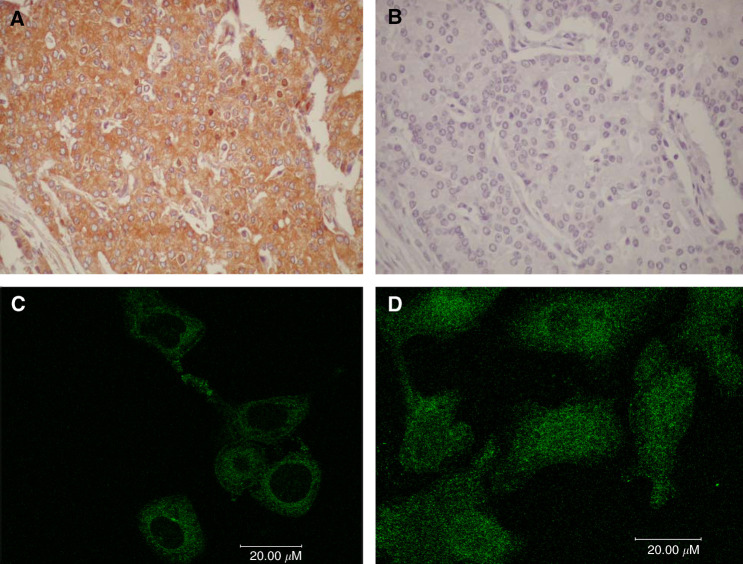
*Upper panels*. Immnunohistochemical stain for p65 in prostate cancer tissues. (**A**) Prostate cancer staining with the antibody to NF-*κ*B/p65 (C-20, sc-372, Santa Cruz) dilution 1 : 180, magnification × 400. (**B**) Immunoreactivity was blocked by preabsortion of the primary antibody with the antigen peptide (sc-372 P, Santa Cruz), magnification × 400. *Lower panels.* Immunofluorescence stain for p65 prostate cancer cells (PC-3). (**C**) Inmunofluorescence staining with the antibody to NF-*κ*B p65 (C-20, Santa Cruz, dilution 1 : 180) in nonstimulated human prostate cancer cells (PC-3) shows only cytoplasmic staining. (**D**) PC-3 cells stimulated with TNF-*α* 100 ng ml^−1^ for 10 min exhibit nuclear staining.

To confirm the specificity of the anti-p65 antibody (C-20, Santa Cruz) used in this study, immunoreactivity was blocked by preabsortion with a specific antigen peptide. This resulted in abrogation of tissue staining (Figure 1A and B). In addition, to assess the ability of the antibody to detect p65 nuclear translocation, we performed an immunofluorescence assay in cultured cells under controlled conditions (Figure 1C and D). Human PC-3 prostate cancer cells were treated with TNF-*α*, a classical activator of p65/NF-*κ*B. In the absence of TNF-*α*, cells exhibited prominent cytoplasmic staining of NF-*κ*B while nuclear staining was not detected. Following a short course of TNF-*α*, the nuclei of tumour cells became intensely stained with the anti-p65 antibody indicating the translocation of NF-*κ*B from the cytoplasm to the nucleus. Finally, Western blot experiments performed as previously reported (Albanell *et al*, 2001) confirmed that the antibody detected a band of approximately 65 kDa, which corresponds to the molecular weight of p65 (data not shown).

Staining patterns (i.e. nuclear and/or cytoplasmic p65) between duplicate cores were concordant in all the specimens (*n*=102) for which duplicates were assessable. Only in three cases (2.9%), one of the cores was not available for analysis. To further validate the data obtained in the tissue microarray, we compared the IHC results of NF-*κ*B/p65 protein staining on whole tissue sections of 14 specimens with the results observed on core biopsies represented in the microarray. All 14 specimens showed the same NF-*κ*B cytoplasmic and nuclear expression patterns, whether assayed in the microarray cores or in the whole tissue sections (data not shown), thus supporting the data generated in the microarray.

**Figure 2:**
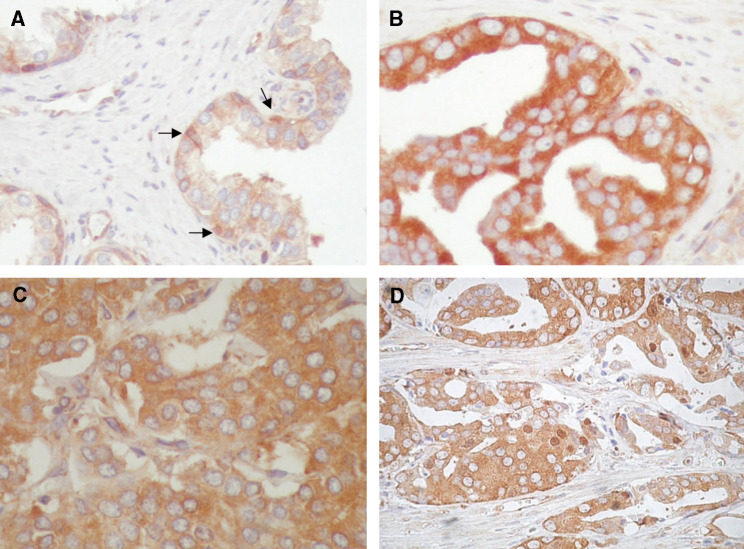
Patterns of NF-*κ*B immunoreactivity in prostate tissues (diaminobenzidine with hematoxylin counterstaining; magnification, × 400). (**A**) Cells of normal prostate glands with weak cytoplasmic NF-*κ*B staining while nuclear NF-*κ*B staining was only seen in scattered basal cells (*arrows*). (**B**) High-grade prostate intraepithelial neoplasia with intense cytoplasmic NF-*κ*B staining. No nuclear immunoreactivity was seen in cells of PIN lesions. (**C**) Prostate cancer specimen with diffuse cytoplasmic NF-*κ*B staining but no nuclear immunoreactivity. (**D**) Prostate cancer specimen with both cytoplasmic and nuclear NF-*κ*B staining in tumour cells.

Normal prostate luminal epithelial cells typically showed NF-*κ*B staining restricted to the cytoplasm (Figure 2A). This cytoplasmic staining was of weak to moderate intensity. In contrast, nuclear NF-*κ*B staining was identified in scattered basal cells of normal glands and was undetected in luminal cells. We also analysed NF-*κ*B expression in high-grade PIN lesions, which are considered as precursors of prostate cancer (Ashida *et al*, 2004; Bostwick and Qian, 2004). The 13 PIN specimens showed cytoplasmic immunoreactivity of strong intensity in the vast majority of epithelial cells. No nuclear immunoreactivity was observed in PIN lesion (Figure 2B). The cytoplasmic staining of cells in PIN was of stronger intensity than that of histologically normal prostatic epithelial cells. The high epithelial density that characterises PIN lesions sometimes resulted in an overlap of cytoplasmic NF-*κ*B staining that may be misinterpreted as some degree of nuclear staining (ie apical snout). However, on careful pathological review, nuclear staining in PIN lesions was undetected.

We also analysed 86 PACs from patients whose clinico-pathological data and follow-up were available (Table 1). Prostate cancer tissues showed variable patterns of NF-*κ*B cytoplasmic and nuclear immunostaining (Figure 2C and D). Cytoplasmic immunoreactivity for NF-*κ*B was negative (ie undetected) in 29 (33.7%) PAC specimens and positive in 57 (66.3%). The scored intensities of cytoplasmic immunoreactivity was + in 21 (24.4%) specimens, ++ in 32 (37.2%) and +++ in 3 (3.4%). Nuclear factor-*κ*B nuclear staining was negative in 39 (45.3%) specimens and positive in 47 (54.7%). The scored intensities of nuclear immunoreactivity was + in 22 (25.6%) specimens, ++ in 15 (17.4%) and +++ in 10 (11.6%). No correlation between cytoplasmic and nuclear NF-*κ*B staining was observed (*χ*^2^ test *P*=0.19). In total, 16 (18.6%) PACs were negative for both nuclear and cytoplasmic NF-*κ*B, 23 (26. 8%) were nuclear negative/cytoplasmic positive, 13 (15.1%) were nuclear positive/cytoplasmic negative and 34 (39.5%) were both nuclear and cytoplasmic positive. Several PAC specimens were scored as negative for both nuclear and cytoplasmic NF-*κ*B. Although these specimens might lack the p65 subunit of NF-*κ*B, it is also possible that under the immunohistochemical assay conditions used, the level of expression was below the limits of detection. No significant correlations were observed between nuclear or cytoplasmic staining for NF-*κ*B and pathologic stage of the disease, Gleason score, preoperative serum PSA levels, surgical margins and seminal vesicle invasion. In more detail, no statistically significant correlations were observed between the percentage of cells with nuclear NF-*κ*B staining and the following factors: Gleason score (Spearman, *P*=0.79), PSA (Spearman, *P*=0.34), stage (Spearman, *P*=0.29), seminal vesicle invasion (*t-*test, *P*=0.10), lymph node status (*t*-test, *P*=0.86) or surgical margins (*t*-test, *P*=0.26). Similarly, the percentage of cells with cytoplasmic NF-*κ*B staining did not statistically correlate with Gleason (Spearman, *P*=0.07), PSA (Spearman, *P*=0.46), stage (Spearman, *P*=0.18), seminal vesicle invasion (*t*-test, *P*=0.25), lymph node status (*t*-test, *P*=0.87) or surgical margins (*t*-test, *P*=0.58). Furthermore, correlation tests performed analysing the clinico-pathologic parameters as binarised continuous variables also lacked statistically significance (Table 2). Since specimens of normal prostate or high-grade PIN lesions were from patients that also had prostate cancer, we assessed whether there was any particular pattern of nuclear NF-*κ*B staining in the corresponding tumours. Tumour cells showed nuclear NF-*κ*B staining in three out of the six patients for whom normal prostate was also assessed and in eight out of the 13 patients for whom high-grade PIN lesions were also assessed. These observations suggest that nuclear translocation of NF-*κ*B occurs during the malignant transformation of the prostate.

### NF-*κ*B cytoplasmic and nuclear expression and disease recurrence

**Figure 3:**
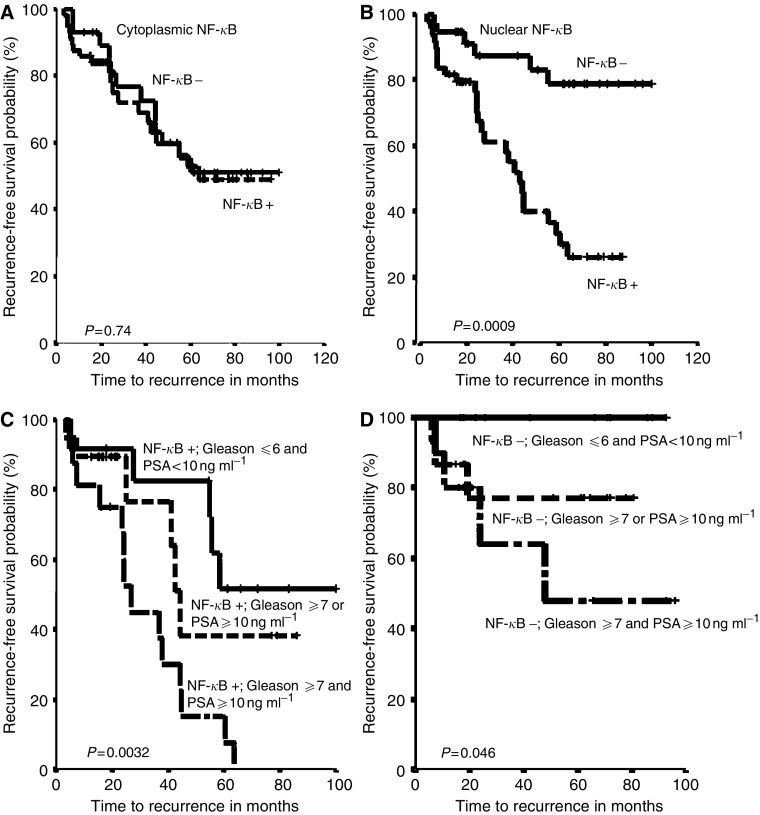
*Upper panels*, Kaplan–Meier survival curves for cytoplasmic and nuclear NF-*κ*B expression in prostate adenocarcinomas. (**A**) No association between NF-*κ*B cytoplasmic expression and biochemical disease recurrence was detected (*P*=0.74). (**B**) Nuclear expression of NF-*κ*B was associated to the chance of biochemical disease-free survival. Patients with nuclear NF-*κ*B had a higher risk of biochemical disease recurrence compared with those whose primary tumours did not have nuclear NF-*κ*B (*P*=0.0009). *Lower panels*, Kaplan–Meier survival curves for NF-*κ*B nuclear expression combined with presurgical serum prostate specific antigen (PSA) levels and Gleason score. (**C**) Patients with positive nuclear NF-*κ*B expression and both (PSA⩾10 ng ml^−1^ and gleason ⩾7) adverse prognostic factors had a greater chance of biochemical disease recurrence than those who presented one (PSA⩾10 ng ml^−1^ or gleason ⩾7) or no (PSA⩽10 ng ml^−1^ and gleason⩽6) adverse independent prognostic factors (*P*=0.0032). (**D**) Patients with negative nuclear NF-*κ*B expression, PSA<10 ng ml^−1^ and Gleason⩽6 presented had a better prognosis compared to patients with negative nuclear NF-*κ*B expression and one (PSA⩾10 ng ml^−1^ or gleason⩾7) or both (PSA⩾10 ng ml^−1^ and gleason⩾7) adverse independent prognostic factors (*P*=0.046).

The relationship between NF-*κ*B expression and the risk of biochemical relapse was assessed using Kaplan–Meier disease outcome analysis. Using a bimodal approach, the presence or absence of NF-*κ*B cytoplasmic staining was not associated with the risk of biochemical relapse of the disease (Figure 3A; log-rank test, *P*=0.74). Using a semiquantitative scoring assessment approach (0, +, ++, +++), cytoplasmic NF-*κ*B expression was similarly not significantly associated with the risk of biochemical relapse (data not shown).

In contrast, NF-*κ*B nuclear staining was a prognostic factor for biochemical relapse of the disease (Figure 3B). Patients with positive nuclear NF-*κ*B expression had a median time to biochemical relapse of the disease of 44 months, while tumours lacking NF-*κ*B nuclear staining did not reach median time to biochemical relapse. The actuarial 5-year biochemical relapse-free survival was 34% in patients with positive nuclear NF-*κ*B staining and 78% patients with negative nuclear NF-*κ*B (Figure 3B; log-rank test, *P*=0.0009). Using a semiquantitative scoring assessments (0, +, ++, +++) approach did not further improve the prognostic value of nuclear NF-*κ*B (data not shown). Kaplan–Meier survival curves for biochemical relapse and log-rank test comparisons also showed that high PSA preoperative levels (*P*=0.0001), high Gleason score (*P*=0.0056) and positive surgical margins (*P*=0.02) were also associated with a higher risk of relapse in log-rank test analysis. Patients with advanced stage also had a worse prognosis than patients with early stage disease, although the differences were not statistically different (*P*=0.23).

On multivariate analysis, positive nuclear NF-*κ*B staining (*P*=0.002), Gleason score (*P*=0.03) and preoperative PSA serum levels (*P*=0.02) were independent prognostic factors for biochemical relapse when all the analysed variables were assessed as binarised variables (Table 3). A second multivariate analysis was performed using nuclear NF-*κ*B, cytoplasmic NF-*κ*B, PSA serum levels, Gleason score and Stage as continuous variables, and surgical margins, seminal vesicle invasion and lymph node status as qualitative variables. In this multivariate analysis, NF-*κ*B nuclear staining (*P*=0.003) and Gleason score (*P*=0.008) were the variables found as independent prognostic factors for biochemical relapse (Table 3). Since nuclear NF-*κ*B was an independent factor, we then analysed the recurrence curves for patients with positive NF-*κ*B nuclear staining stratified by the presence or absence of high Gleason score and/or high preoperative PSA. Notably, all tumours featuring the presence of all three adverse prognostic factors experienced biochemical relapse of the disease. Tumours with nuclear NF-*κ*B expression and no additional risk factors (ie low Gleason and low preoperative PSA) had the lowest rate of biochemical recurrence in the nuclear NF-*κ*B positive group. Tumours with positive NF-*κ*B nuclear staining and only one of the two additional adverse prognostic factors (ie high Gleason or high preoperative PSA, but not both) had an intermediate rate of biochemical disease relapse (Figure 3C; log-rank test, *P*=0.0032). Finally, the incidence of biochemical disease recurrence in tumours with no nuclear NF-*κ*B staining varied according to the presence or absence of high preoperative PSA or high tumour grade. In tumours that were devoid of nuclear NF-*κ*B expression and were also low grade and featured a low preoperative serum PSA level, 0 (0%) experienced biochemical relapse during the available follow-up period (Figure 3D; log-rank test, *P*=0.046).

## DISCUSSION

In the present study, we provide novel data that strongly point to NF-*κ*B nuclear translocation (ie activation) as a late event in prostate cancer development and also that nuclear NF-*κ*B expression is an independent predictor of a significantly increased risk of biochemical (PSA) relapse in patients that underwent prostatectomy. Furthermore, a combined prognostic analysis taking into account preoperative PSA levels, Gleason grade and nuclear NF-*κ*B staining could identify subsets of patients with markedly different risks of biochemical relapse.

In histologically normal prostatic glands, NF-*κ*B expression was generally weak and located in the cytoplasm of the luminal epithelial cells whereas nuclear staining was only seen in scattered basal cells. In PIN lesions, NF-*κ*B was exclusively overexpressed in the cytoplasm and nuclear NF-*κ*B was not detected. For PAC, NF-*κ*B expression and its subcellular localisation were highly variable among different specimens. There was no correlation between NF-*κ*B nuclear and cytoplasmic staining. Since PAC, PIN and histologically normal specimens came from the same patients and staining patterns differed, this evokes an apparent lack of any detectable paracrine mechanism of NF-*κ*B activation.

The molecular determinants of the diverse patterns in NF-*κ*B expression and subcellular localisation in prostate cancer deserve further studies. The current observations suggest that in the early stages of prostate tumorigenesis (i.e. in PIN lesions) (Sasaki *et al*, 2001; Yu *et al*, 2003), cytoplasmic NF-*κ*B levels increase by as yet unknown mechanisms and nuclear translocation of NF-*κ*B does not occur until invasive cancer develops. Along this line, it has been shown recently that the majority of prostate cancer lymph node metastasis had evident nuclear NF-*κ*B staining (Ismail *et al*, 2004). These findings in prostate tumorigenesis are consistent with previous observations in other cancer types, such as gastric (Sasaki *et al*, 2001), uterine cervix (Nair *et al*, 2003), colorectal (Yu *et al*, 2003), breast (Biswas *et al*, 2004), and head and neck squamous cell cancers (Nakayama *et al*, 2001), showing that NF-*κ*B activation occurs mainly in established cancer tissues rather than in normal or premalignant lesions.

In the present study, NF-*κ*B staining was not significantly linked to other known prognostic factors of biochemical relapse such as preoperative PSA levels, surgical margins or Gleason grade. Others have reported similar findings, thus suggesting that NF-*κ*B expression may add to current established prognostic factors in prostate cancer (Ismail *et al*, 2004). Along this line, in the present series, immunohistochemical nuclear NF-*κ*B staining/activation in prostate cancer was an independent prognostic factor of biochemical relapse for prostate cancer patients. We found statistically significant differences in biochemical free-survival as a function of NF-*κ*B expression, suggesting the potential biological relevance of this marker. PSA relapse occurred typically months or years after prostatectomy and, therefore, the possibility of NF-*κ*B nuclear activation and PSA increase favored by inflammatory stimulus in the prostate gland, can be excluded. Although the shortest follow-up was of 12.6 months (a single patient), the mean follow-up of the series was 57 months and allowed to have a number of relapses high enough to detect outcome differences. Patients with nuclear NF-*κ*B staining had a hazard ratio for biochemical relapse five-fold higher than patients with no NF-*κ*B nuclear staining. This prognostic significance of NF-*κ*B in PACs reveals that the biologically active/nuclear form of NF-*κ*B is linked to outcome.

A prior study showed that total overexpression of NF-*κ*B by IHC on prostatectomy specimens was as an independent prognostic factor in human prostate cancer (Ross *et al*, 2004). Since it is extensively considered that cytoplasmic NF-*κ*B is an inactive form with nuclear translocation required for activation, nuclear staining was also specifically analysed in that study. However, NF-*κ*B nuclear localisation was unrelated to the risk of biochemical relapse (Ross *et al*, 2004). Notably, the rate of nuclear NF-*κ*B staining (15%) in the study by Ross *et al* was lower than what we report in the present study (54.7%) or other reports (Fradet *et al*, 2004). We showed that the antibody used in the present work detected nuclear translocation (activation) of NF-*κ*B/p65 under controlled experimental cell culture conditions. In prostate cancer cells stimulated with TNF-*α*, the antibody exhibited strong nuclear staining, as assayed by immunofluorescence, while in nonstimulated cells the staining pattern was mainly cytoplasmic. Specificity of the antibody to p65 was further shown by Western blot and by preabsorption with a specific blocking peptide by IHC. Therefore, the current study confirms the prior finding of a link between NF-*κ*B/p65 and prostate cancer outcome and extends the observation by showing that nuclear localisation of NF-*κ*B/p65 (the biologically active form) is specifically associated to outcome while cytoplasmic staining lacked prognostic value.

Other studies also support the concept that nuclear NF-*κ*B localisation is related to outcome. In another study, nuclear NF-*κ*B was found in 40% of prostate tumours in 40 specimens assayed (Lessard *et al*, 2003). As in the current study, nuclear NF-*κ*B did not significantly correlate with Gleason score. In a highly selected series of 30 patients, nuclear localisation was significantly associated with the prognostic groups (Lessard *et al*, 2003). Multivariate analysis was not reported in that study. A study published recently found nuclear NF-*κ*B expression as an independent prognostic marker of eventual biochemical recurrence in a series of 42 men, all of them with positive surgical margins after radical prostatectomy. In that study, NF-*κ*B was measured at the positive surgical margins and 64% of the specimens were scored as NF-*κ*B positive. In a multivariate analysis, nuclear NF-*κ*B staining was an independent prognostic factor in this population (Fradet *et al*, 2004). In our series reported here, 54 (62.7%) patients did not have positive surgical margins. Of note, the percentage of nuclear NF-*κ*B staining was not associated with the presence or absence of pathologically confirmed surgical margins. In our multivariate analysis, nuclear NF-*κ*B expression was confirmed as an independent prognostic marker. Hence, this result provides novel data by showing that nuclear localisation of NF-*κ*B has an independent prognostic role in PAC patients that underwent surgery (also regardless of the pathological state of the surgical margins). Moreover, a total of five patients had positive lymph nodes at the time of prostatectomy. NF-*κ*B was not assayed in these metastatic nodes. However, the five patients had nuclear NF-*κ*B staining in the corresponding primary prostate cancers, thus further suggesting a possible link between NF-*κ*B and a metastatic phenotype.

The above studies and the present data support a role for NF-*κ*B in the clinical behaviour of prostate cancer. It should be noted, however, that this field is in an early stage of development and differences in the IHC methods, scoring interpretations or patient populations could explain the different rates of expression and subcellular localisation of NF-*κ*B among studies. In this regard, however, we should point out that one of us (JSR) reviewed the scorings of the current and also a previously published series (Ross *et al*, 2004), thus reducing the possibility of heterogeneous criteria for nuclear scoring between these two studies. Collectively, these two studies concur on a role for NF-*κ*B expression in prostate cancer clinical behaviour and the current one suggests that the nuclear (activated) form of p65 is the one that is specifically linked with a higher likelihood of relapse.

To date, positive margins after prostatectomy, PSA serum levels prior to prostatectomy, combined Gleason score and tumour node metastasis (TNM) staging are considered prognostic factors for prostate cancer progression (Baldwin, 1996; Karin *et al*, 2004). In the current series, preoperative PSA levels, Gleason score and positive margins were significantly associated to the risk of biochemical relapse by Kaplan–Meier analysis. With the goal of building on these prognostic factors, we combined nuclear NF-*κ*B staining results with PSA preoperative levels and Gleason scores, which were the factors that retained significant prognostic value on multivariate analysis. Among these three factors, nuclear NF-*κ*B expression was the more strongly associated to outcome. Promising results emerged from such analysis since patients with all three adverse factors (nuclear NF-*κ*B, high PSA and high Gleason) experienced a biochemical relapse, while all patients with none of these adverse factors (i.e. undetected nuclear NF-*κ*B, low PSA and low Gleason) were free of biochemical recurrence. A prior study with 30 patients that combined nuclear staining with Gleason grade also improved prediction of clinical outcome (Lessard *et al*, 2003). These observations merit further analysis in prospective studies given its potential interest in adjuvant management strategies of patients with PAC that undergo prostatectomy.

Our findings indicate a significant relationship between p65/NF-*κ*B nuclear staining and biochemical recurrence. However, the functional relevance of this immunoreactivity on NF-*κ*B activation is yet unknown. This limitation is based on the fact that p65/NF-*κ*B nuclear translocation is necessary but not sufficient for NF-*κ*B-induced transcriptional activity, since both recruitment of NF-*κ*B to target genes and NF-*κ*B-induced transcriptional events after recruitment are needed for this to occur (Natoli *et al*, 2005). It should be also noted that the minimum percentage of tumour cells with nuclear p65 staining required to potentially result in detectable NF-*κ*B-induced transcriptional activity remains uncharacterised. Another limitation of using p65/NF-*κ*B as a marker of activation is that noncanonical pathways, independent of p65, might also activate NF-*κ*B.

A previous study demonstrated that NF-*κ*B regulates the transcription of PSA, which is a marker of prostate cancer progression. Furthermore, the authors also showed that androgen-independent prostate cancer xenografts had higher constitutive that NF-*κ*B binding activity than their androgen-dependent counterparts. These elegant preclinical studies suggested a role of NF-*κ*B in prostate cancer progression (Chen and Sawyers, 2002) and our present work extends this view to a clinical setting. Moreover, in prostate cancer cells, constitutive activation of NF-*κ*B has been associated to the activation of signalling transduction pathways involving tyrosine kinases and, more specifically, molecules such as Rho-A, Ki-Ras or PTEN/Akt (Gasparian *et al*, 2002; Li and Sarkar, 2002; Kim *et al*, 2002; Mayo *et al*, 2002; Hodge *et al*, 2003). Transforming growth factor *β*1 and *β*2 have been also involved in NF-*κ*B activation in prostate cancer cells (Park *et al*, 2003; Lu *et al*, 2004). These pathways appear to mediate NF-*κ*B activation as a result of their effect on NF-*κ*B inducing kinase, and I*κ*B kinase activation (IKK) (Gasparian *et al*, 2002; Suh *et al*, 2002). It is well known that in quiescent cells, NF-*κ*B is bound to I*κ*B inhibitory proteins and retained in the cytoplasm. However, phosphorylation of I*κ*Bs by IKK results in the rapid degradation of I*κ*B proteins by the proteasome, freeing p65/NF-*κ*B to enter the nucleus, bind to DNA, and activate transcription. I*κ*B expression in prostate cancer has been assessed recently (Ross *et al*, 2004). In that study, decreased expression of I*κ*B*α* in primary prostate tumours correlated only with tumour grade. However, contrarily to what might be expected, I*κ*B*α* expression was not inversely related to p65/NF-*κ*B. Clearly, there is much work needed to assess the interplay between the multiple members of the IKK/NF-*κ*B family. Along this line, an study in endometrial cancer has shown that nuclear immunostaining for members of the NF-*κ*B family correlated with negativity for members of the I*κ*B family in some cases (Pallares *et al*, 2004). It will be of importance to characterise whether these molecules, or additional ones that may be shown of importance in the future, are also linked to NF-*κ*B activation in human prostate cancers. In addition to its role in prostate cancer behaviour (Andela *et al*, 2003), NF-*κ*B activation is also implicated in chemo- and radioresistance. These observations are complemented by studies showing that NF-*κ*B inhibition is a promising strategy for prostate cancer treatment, and, particularly, as a chemo- or radio-sensitisation strategy (Palayoor *et al*, 1999; Altuwaijri *et al*, 2003; Flynn *et al*, 2003; Kikuchi *et al*, 2003; Chendil *et al*, 2004). Clearly, inhibition of NF-*κ*B may potentiate the antineoplastic effect of conventional chemotherapeutic agents (Sanchez-Perez *et al*, 2002).

In addition to the studies on the expression of NF-*κ*B in human PACs discussed above, there are data using interleukin(IL)-6 as a surrogate marker of NF-*κ*B activation in patients with prostate cancer that further suggest a role of NF-*κ*B in a clinical setting (Zerbini *et al*, 2003). Along this line, high serum levels of IL-6 in PAC patients have been linked to disease progression, hormone-independence and chemotherapy resistance (Nakashima *et al*, 2000; Sanchez-Perez *et al*, 2002; Michalaki *et al*, 2004). Also, dexamethasone, a glucocorticoid used commonly for prostate cancer treatment, disrupts the NF-*κ*B-IL-6 pathway and this is thought to mediate the antitumour effect (Nishimura *et al*, 2001). Finally, recent evidence in a phase I clinical trial has suggested that the proteasome inhibitor bortezomib has activity against human prostate cancer and reduces the expression of serum IL-6 and PSA levels in some patients (Papandreou and Logothetis, 2004). This is relevant here since the degradation of the inhibitor of NF-*κ*B, I*κ*B, is dependent on the ubiquitin–proteasome pathway, and proteasome inhibition results in inhibition of NF-*κ*B (Elliott and Ross, 2001; Adams, 2004; Papandreou and Logothetis, 2004).

Based on the preclinical data and the emerging clinical results, NF-*κ*B appears to be a potential important prognostic factor and/or target of therapy in human prostate cancer. The current study shows that NF-*κ*B activation occurs in the transition from a preneoplastic state to prostate cancer and that NF-*κ*B activation is a molecular marker that independently predicts a high risk of biochemical relapse of prostate cancer. These data support the concept of NF-*κ*B inhibition as an attractive research strategy for prostate cancer treatment.

## Figures and Tables

**Table 1:** Clinicopathological characteristics of the patients with prostate adenocarcinomas (*n*=86)

Mean age	66 years (range, 45–79 years)
Mean follow-up time	57 months (range, 12.6–100 months)
Mean PSA level	10.92 ng ml^−1^ (range, 0.61–34 ng ml^−1^)
	
*PSA*	
<10 ng ml^−1^	51 patients (59.3%)
⩾10 ng ml^−1^	35 patients (40.7%)
	
*Gleason grade*	
⩽6	35 patients (40.7%)
⩾7	51 patients (59.3%)
	
*Stage*	
Organ confined (T2a,T2b)	51 patients (59.3%)
T2a	14 patients (16.3%)
T2b	37 patients (43.0%)
Advanced (T3a,T3b)	35 patients (40.7%)
T3a	22 patients (25.6%)
T3b	13 patients (15.1%)
	
*Surgical margins*	
Negative	54 patients (62.7%)
Positive	32 patients (37.3%)
	
*Seminal vesicle invasion*	
Absent	73 patients (84.8%)
Present	13 patients (15.2%)
	
*Lymph node status*	
Negative	81 patients (94.1%)
Positive	5 patients (5.9%)
Biochemical relapse	33 patients (38.3%)
Metastasis	6 patients (6.9%)
Prostate cancer related deaths	3 patients (3.5%)

**Table 2:** Nuclear and cytoplasmic immunoreactivity to NF-*κ*B in PACs specimens and clinicopathological characteristics (*n*=86)

	**Nuclear**		**Cytoplasmic**			
	**NF-*κ*B+(*n*=47)**	**NF-*κ*B−(*n*=39)**	***P***	**NF-*κ*B+(n=57)**	**NF-*κ*B−(*n*=29)**	***P***
High grade^a^ (*n*=51)	23/47 (48.9%)	28/39 (73.7%)	0.56	35/57 (61.4%)	16/29 (55.2%)	0.56
Advanced Stage^a^ (*n*=35)	19/47 (40.4%)	16/39 (41.0%)	0.30	22/57 (38.6%)	13/29 (44.8%)	0.57
Positive margin^a^ (*n*=32)	12/47 (25.5%)	20/39 (51.3%)	0.14	23/57 (40.3%)	9/29 (31.0%)	0.39
Mean±s.d. PSA^b^	9.77±6.25	11.78±7.64	0.29	10.65±6.35	11.45±8.47	0.62
Presence of seminal vesicle invasion (*n*=13)	6/47 (12.7%)	7/39 (17.9%)	0.50	5/57 (8.7%)	8/29 (27.8%)	0.61
Positive lymph-nodes^c^ (*n*=5)	5/47 (10.6%)	0/39 (0%)	0.06	2/57 (3.5%)	3/29 (10.3%)	0.20

a *χ*^2^ test.

b Student's *t* test.

c Fisher's exact test. For Spearman's correlation tests, see text.

**Table 3:** Multivariate Cox proportional hazards model analysis of prognostic factors for biochemical relapse in prostate cancer patients

	**Hazard ratio**	**95% confidence interval**	***P* value**
*Stage*			0.91
Organ confined (T2a, T2b)	1.0	Reference	
Advanced stage (T3a, T3b)	1.66	0.37–3.00	
			
*PSA* a			0.02
<10 ng ml^−1^	1.0	Reference	
⩾10 ng ml^−1^	2.69	1.15–6.30	
			
*Gleason* a			0.03
⩽6	1.0	Reference	
⩾7	2.85	1.10–7.43	
			
*Surgical margins*			
Negative	1.0	Reference	0.66
Positive	0.81	0.31–2.08	
			
*Seminal invasion*			0.38
Absence	1.0	Reference	
Presence	1.66	0.52–5.23	
			
*Lymph-node status*			0.28
Positive	1.0	Reference	
Negative	0.44	0.99–1.99	
			
*Cytoplasmic NF-κB*			0.86
Negative	1.0	Reference	
Positive	0.93	0.40–2.12	
			
*Nuclear NF-κB* a			0.002
Absence	1.0	Reference	
Presence	5.00	1.84–13.55	

a In a multivariate analysis including PSA, Gleason, stage, cytoplasmic NF-*κ*B and nuclear NF-*κ*B as continuous variables, *P*-values were 0.8 for PSA; 0.008 for Gleason; and 0.003 nuclear NF-*κ*B. See text for additional details.
